# Supplementary material for: The endothelial scavenger receptor stab2 is required for proper hematopoietic stem and progenitor cell development in the fetal blood stem cell niche
Source: bioRxiv. 2026 Jul 23:2026.07.07.737122. Originally published 2026 Jul 8. Preprint. [Version 2] doi: 10.64898/2026.07.07.737122 (PMC13370947; doi:10.64898/2026.07.07.737122)
Supplement: Supplement 1 [file media-1.pdf]

Supplemental information - Document S1

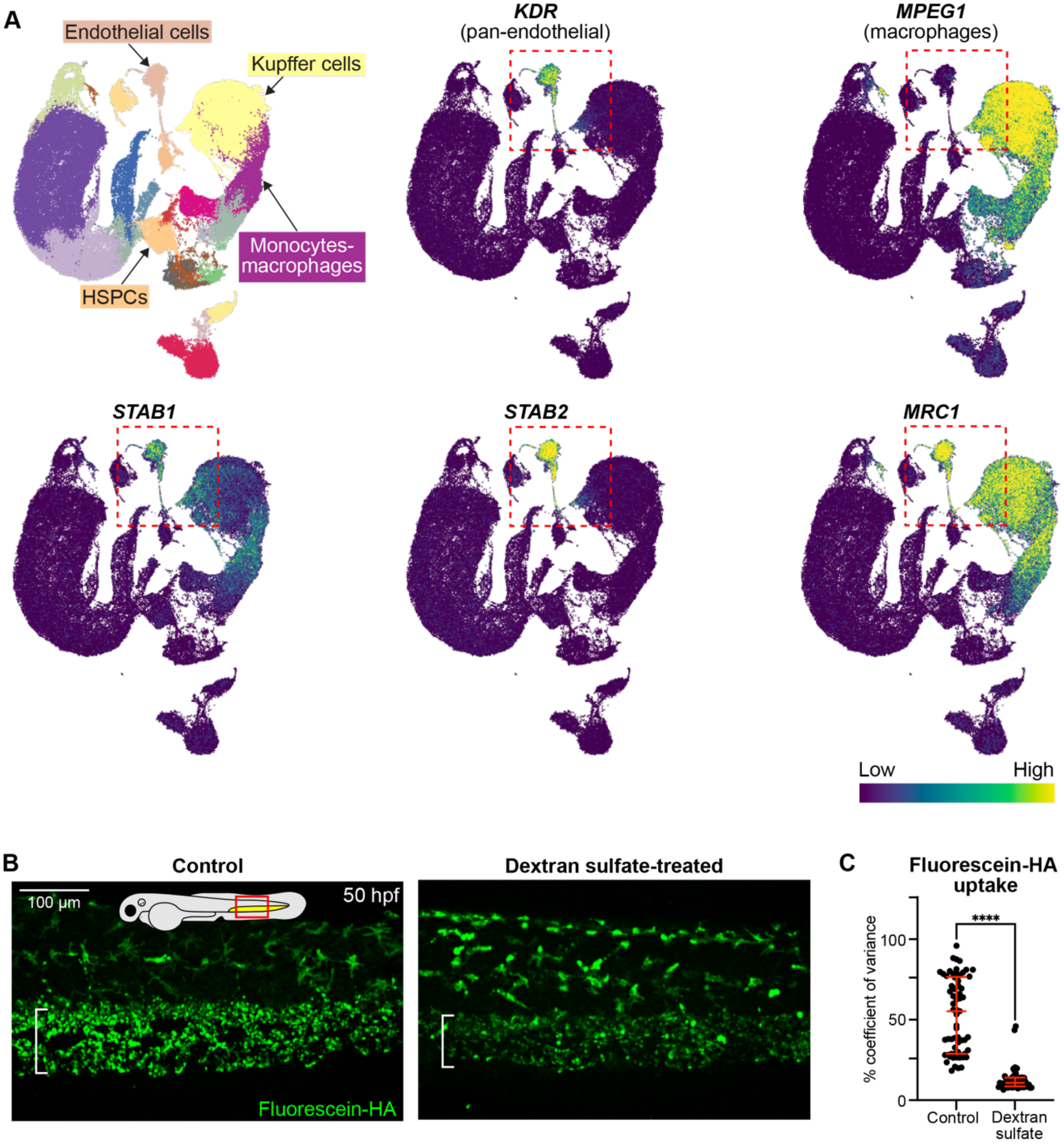

Figure S1

Figure S1. Uncropped UMAPs for human fetal liver scRNA-seq data and quantification of HA uptake in the zebrafish CHT. A. UMAP of human fetal liver scRNA-sequencing from Popescu et al., *Nature* 2019[26] with colors indicating cell type clusters (top left). Other UMAPs are uncropped versions of those shown in Figure 1A, with a red dotted line indicating the cropped region. **B–C.** Representative images (B) and quantification (C) of fluorescein-HA uptake in the CHT of the same embryos shown in Figure 1E. Bracket denotes location of CHT in this and all subsequent figures. For dot plots in this and all subsequent figures, each dot represents one zebrafish embryo, unless otherwise noted. Mann-Whitney test, error bars represent median ± interquartile range. ****$p<0.0001$.

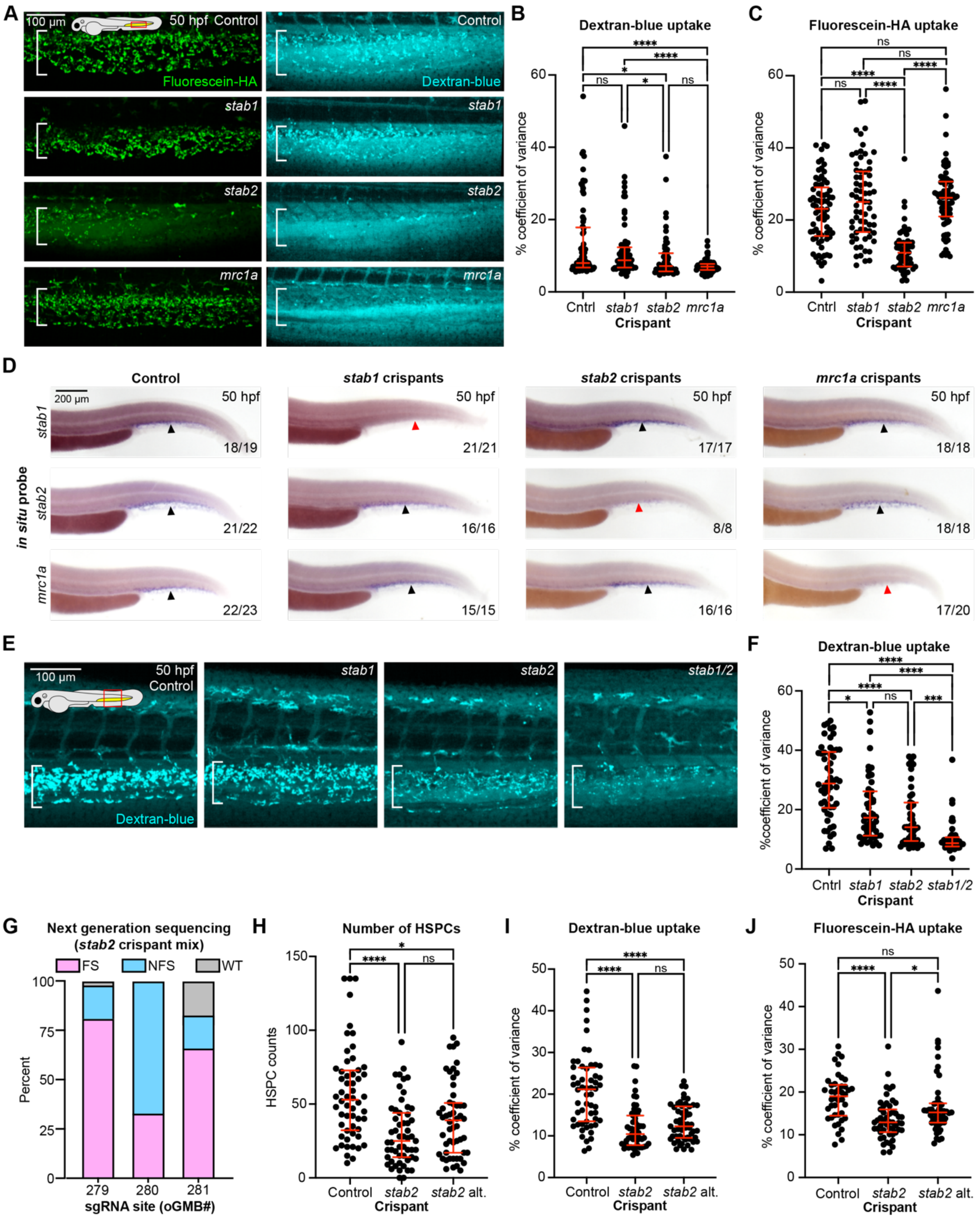

A
100 µm
50 hpf Control
Fluorescein-HA
Control
Dextran-blue
stab1
stab2
mrc1a
B
Dextran-blue uptake
% coefficient of variance
Cntrl stab1 stab2 mrc1a
Crispant
C
Fluorescein-HA uptake
D
Control
stab1 crispants
stab2 crispants
mrc1a crispants
200 µm
50 hpf
in situ probe
18/19
21/21
17/17
18/18
21/22
16/16
8/8
18/18
22/23
15/15
16/16
17/20
E
50 hpf Control
stab1/2
F
%coefficient of variance
Cntrl stab1 stab2 stab1/2
G
Next generation sequencing (stab2 crispant mix)
FS NFS WT
Percent
279 280 281
sgRNA site (oGMB#)
H
Number of HSPCs
HSPC counts
Control stab2 stab2 alt.
Crispant
I
Dextran-blue uptake
J
Fluorescein-HA uptake

Figure S2

Figure S2. Additional characterization of *stab1*, *stab2* and *mrc1a* crispants. A–C. Representative images (A) and quantification of dextran-blue (B) and fluorescein-HA (C) uptake in the CHT of control, *stab1*, *stab2* and *mrc1a* crispants (same embryos as in Figure 2A–B). **D.** Whole-mount *in situ* hybridization (WISH) of *stab1*, *stab2* or *mrc1a* in the indicated crispant background. Black arrowheads indicate staining in the CHT. Red arrowheads indicate reduced staining compared to in controls. The fraction of embryos that exhibit the shown phenotype across one biological replicate is indicated. **E–F.** Representative images (E) and quantification (F) of dextran-blue uptake in the CHT of control, *stab1*, *stab2* and *stab1/2* (double) crispants (same embryos as in Figure 2C–D). **G.** Percentage of frameshift causing (FS) and non-frameshift causing (NFS) CRISPR edits at three out of the four sgRNA cut sites in *stab2* crispants, identified using next generation sequencing. Sequencing across the 4th sgRNA site (not shown) produced low read counts (<100 per embryo) in the majority of embryos. Data are of two pooled biological replicates. **H–J.** Number of *runx1:mCherry* HSPCs (H) and quantification of dextran-blue (I) and fluorescein-HA (J) uptake in control and *stab2* crispants, compared to crispants generated with four different sgRNAs in *stab2* (*stab2* alt), at 50 hpf. The same embryos were quantified in H, I and J. Control embryos for all crispant data are *golden* (*slc24a5*) crispants in this and all subsequent figures, unless otherwise noted. Kruskal-Wallis test, error bars represent median ± interquartile range. *p<0.05, ***p<0.001, ****p<0.0001, n.s., not significant.

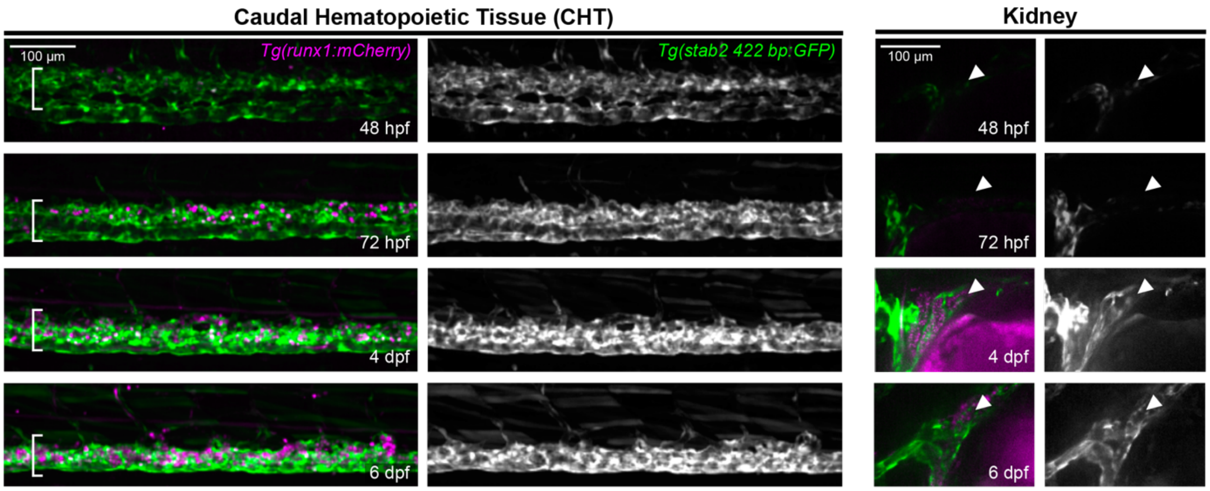

Figure S3

Figure S3. A *stab2* enhancer transgene is expressed by ECs of the embryonic and developing adult hematopoietic niches.

Representative images of *stab2* enhancer GFP and *runx1:mCherry* transgenes in the CHT (left) and larval kidney (right). GFP is shown in grayscale. White arrowheads indicate expression of the *stab2* enhancer GFP transgene in the kidney.

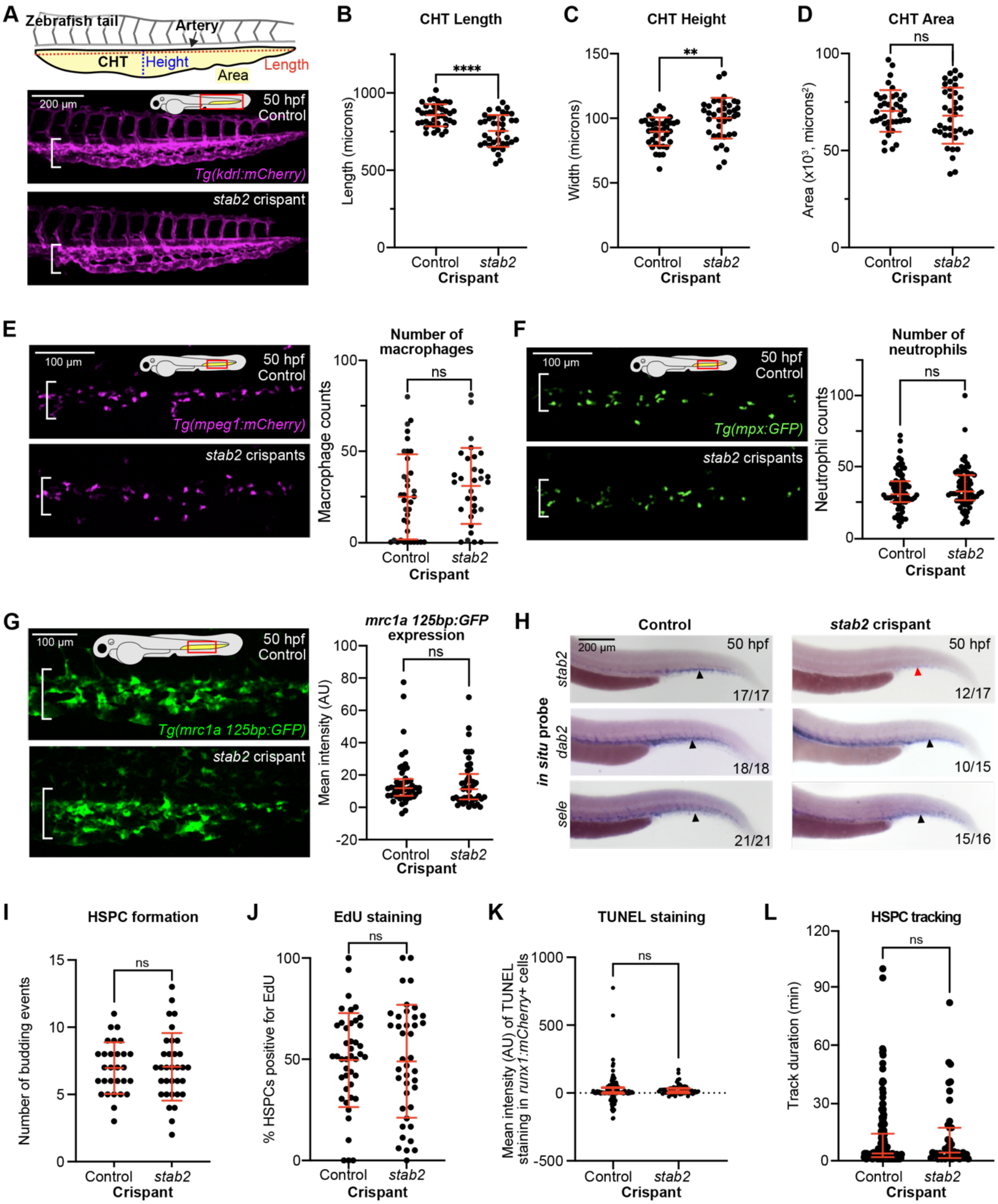
A
Zebrafish tail
Artery
CHT
Height
Area
Length
200 µm
50 hpf
Control
Tg(kdrl:mCherry)
stab2 crispant
B
CHT Length
****
Length (microns)
1000
500
0
Control
stab2
Crispant
C
CHT Height
**
Width (microns)
150
100
50
0
Control
stab2
Crispant
D
CHT Area
ns
Area (x10³, microns²)
100
50
0
Control
stab2
Crispant
E
100 µm
50 hpf
Control
Tg(mpeg1:mCherry)
stab2 crispants
Number of macrophages
ns
Macrophage counts
100
50
0
Control
stab2
Crispant
F
100 µm
50 hpf
Control
Tg(mpx:GFP)
stab2 crispants
Number of neutrophils
ns
Neutrophil counts
100
50
0
Control
stab2
Crispant
G
100 µm
50 hpf
Control
Tg(mrc1a 125bp:GFP)
stab2 crispant
mrc1a 125bp:GFP expression
ns
Mean intensity (AU)
100
80
60
40
20
0
-20
Control
stab2
Crispant
H
Control
stab2 crispant
200 µm
50 hpf
50 hpf
in situ probe
stab2
dab2
sele
17/17
12/17
18/18
10/15
21/21
15/16
I
HSPC formation
ns
Number of budding events
15
10
5
0
Control
stab2
Crispant
J
EdU staining
ns
% HSPCs positive for EdU
100
50
0
Control
stab2
Crispant
K
TUNEL staining
ns
Mean intensity (AU) of TUNEL staining in runx1:mCherry+ cells
1000
500
0
-500
Control
stab2
Crispant
L
HSPC tracking
ns
Track duration (min)
120
90
60
30
0
Control
stab2
Crispant

Figure S4

Figure S4. Tissue structure, cellular composition and endothelial gene expression within the CHT remain intact in *stab2* crispants. A. Schematic indicating CHT length, height and area measurements in the tail of the zebrafish embryo with representative images below. **B–D.** Graphs report the length (B), height (C) and area (D) of the CHT vascular plexus in 50 hpf crispants as depicted in A. **E–F.** Representative images (left) and quantification (right) of *mpeg1:mCherry*(+) macrophage (E) and *mpx:GFP*(+) neutrophil (F) numbers in the CHT of *stab2* crispants and controls. **G.** Representative images (left) and background-subtracted mean intensity (plotted, right) of a CHT EC-specific transgene, *mrc1a 125bp:GFP* in *stab2* and control crispants. **H.** WISH of niche EC genes selectively expressed in the vasculature of the CHT. Black arrowheads indicate staining in the CHT. Red arrowhead indicates reduced staining compared to in the control. The fraction of embryos that exhibit the shown phenotype across two pooled biological replicates is indicated. **I.** Number of nascent *cd41:GFP*(+) HSPCs that budded from the aorta-gonad-mesonephros region from 36–42 hpf in control embryos (uninjected) and *stab2* crispants. **J.** Percentage of *runx1:mCherry*(+) HSPCs positive for EdU staining in fixed embryos at 60 hpf (exposed to EdU from 48–60 hpf). **K**. Background-subtracted mean intensity of TUNEL staining in *runx1:mCherry*(+) HSPCs in 50 hpf fixed embryos (two pooled biological replicates). **L.** Track durations of *runx1:mCherry*(+) HSPCs during a 2 hr timelapse from ~52–54 hpf. In the dot plots in K and L, each dot represents one *runx1:mCherry*(+) HSPC (number of embryos for L: n = 9 for controls, n = 7 for *stab2* crispants; number of embryos for K: n = 25 for controls, n = 11 for *stab2* crispants). For B–D, E, I and J, Unpaired student's t-test, error bars represent mean ± standard deviation. For F, G, K, and L, Mann-Whitney test, error bars represent median ± interquartile range. $^{**}p<0.01$, $^{****}p<0.0001$, n.s., not significant.

Table S1| Olignonucleotides

Unique Identifier	Description	Category	Sequence	Source
oGMB1	Universal primer for CRISPR sgRNAs	sgRNA template	AAAAGCACCGACTCGGTGCCACTTTTTCAAGTTGATAACGGACT AGCCTTATTTTAACTTGCTATTTCTAGCTCTAAAAC	This paper
oGMB279	*stab2* guide 1	sgRNA template	taatacgactcactataGGGGCAGATTCTAGTCAATGgttttagagctagaa	This paper
oGMB280	*stab2* guide 2	sgRNA template	taatacgactcactataGGCAACGGCATATGTAAAGAgttttagagctagaa	This paper
oGMB281	*stab2* guide 3	sgRNA template	taatacgactcactataGGAACTGGCAGGTGGCCTATgttttagagctagaa	This paper
oGMB282	*stab2* guide 4	sgRNA template	taatacgactcactataGGAGGTTGCTCAGCCCATGgttttagagctagaa	This paper
oGMB502	*stab1* guide 1	sgRNA template	taatacgactcactataGGGAGGTCCAAAGCAACGGCgttttagagctagaa	This paper
oGMB503	*stab1* guide 2	sgRNA template	taatacgactcactataGGCGATGGACTCTCGTGTTAgttttagagctagaa	This paper
oZSI17	*stab1* guide 3	sgRNA template	taatacgactcactataGGCGATGGCAGACGTTGCTAgttttagagctagaa	This paper
oZSI19	*stab1* guide 4	sgRNA template	taatacgactcactataGGTCGCCAGGTAGAGCTGTCgttttagagctagaa	This paper
oZSI24	*mrc1a* guide 1	sgRNA template	taatacgactcactataGGTCACCCCTCTCTGGAACCgttttagagctagaa	This paper
oZSI25	*mrc1a* guide 2	sgRNA template	taatacgactcactataGGGCTCAAAACATCAAGGACgttttagagctagaa	This paper
oZSI26	*mrc1a* guide 3	sgRNA template	taatacgactcactataGGAGGATTGTGTCCTCATCAgttttagagctagaa	This paper
oZSI27	*mrc1a* guide 4	sgRNA template	taatacgactcactataGGAGTCGCTGGCAGGTGTATgttttagagctagaa	This paper
oGMB538	*stab2* guide 1, alt	sgRNA template	taatacgactcactataGGGTGTCAAGTTCATAAATAgttttagagctagaa	This paper
oGMB539	*stab2* guide 2, alt	sgRNA template	taatacgactcactataGGGAACTGAGATCCTCTGGGgttttagagctagaa	This paper
oGMB540	*stab2* guide 3, alt	sgRNA template	taatacgactcactataGGATACGCTAAGATGCCACCgttttagagctagaa	This paper
oGMB541	*stab2* guide 4, alt	sgRNA template	taatacgactcactataGGATTCGAGAAGTTTGTTGAgttttagagctagaa	This paper
oGMB116	*slc24a5* guide 1	sgRNA template	taatacgactcactataGGCACCGTGAAGAATCCTTCgttttagagctagaa	This paper
oGMB117	*slc24a5* guide 2	sgRNA template	taatacgactcactataGGTGACGGCGGCGACACTGAgttttagagctagaa	This paper
oGMB119	*slc24a5* guide 3	sgRNA template	taatacgactcactataGGTTGACGTGGGCTCCCCGGgttttagagctagaa	This paper
oGMB120	*slc24a5* guide 4	sgRNA template	taatacgactcactataGGGTGTCTCCTGCTGGTGTAgttttagagctagaa	This paper
KE stab2 fw primer	Genotype *stab2(ibl2)*, forward	Genotyping	GCATCAAAACAAATGTAACACAGC	This paper
KE stab2 rv primer	Genotype *stab2(ibl2)*, reverse	Genotyping	ATACACACAGCGGGTAGAGC	This paper
oGMB572	Non-barcoded forward (oGMB279 site)	NGS sequencing	atacataaaagtgatgcccctg	This paper
oGMB573	Barcode 1 reverse (oGMB279 site)	NGS sequencing	TCAGCTAAGGTAAATGGGCTTTATGATGTCACACC	This paper
oGMB574	Barcode 2 reverse (oGMB279 site)	NGS sequencing	TCAGTGAGCGGAAATGGGCTTTATGATGTCACACC	This paper
oGMB575	Barcode 3 reverse (oGMB279 site)	NGS sequencing	TCAGTCTATTCGTATGGGCTTTATGATGTCACACC	This paper
oGMB576	Non-barcoded forward (oGMB280 site)	NGS sequencing	ctggaattcagcatggaaaatgttc	This paper
oGMB577	Barcode 1 reverse (oGMB280 site)	NGS sequencing	TCAGCTAAGGTAActaatacCTTGATCACAATGAATTCC	This paper
oGMB578	Barcode 2 reverse (oGMB280 site)	NGS sequencing	TCAGTGAGCGGAActaatacCTTGATCACAATGAATTCC	This paper
oGMB579	Barcode 3 reverse (oGMB280 site)	NGS sequencing	TCAGTCTATTCGTctaatacCTTGATCACAATGAATTCC	This paper
oGMB580	Non-barcoded forward (oGMB281 site)	NGS sequencing	GTGTGAACCTGTCAATCAGTG	This paper
oGMB581	Barcode 1 reverse (oGMB281 site)	NGS sequencing	TCAGCTAAGGTAAgacataaattaagatgtattacCTGCAGT	This paper
oGMB582	Barcode 2 reverse (oGMB281 site)	NGS sequencing	TCAGTGAGCGGAAgacataaattaagatgtattacCTGCAGT	This paper
oGMB583	Barcode 3 reverse (oGMB281 site)	NGS sequencing	TCAGTCTATTCGTgacataaattaagatgtattacCTGCAGT	This paper
oGMB584	Non-barcoded forward (oGMB282 site)	NGS sequencing	CGGCACATATTGTGAAGgtattc	This paper
oGMB585	Barcode 1 reverse (oGMB282 site)	NGS sequencing	TCAGCTAAGGTAAaaactaactgtagatgtagacattc	This paper
oGMB586	Barcode 2 reverse (oGMB282 site)	NGS sequencing	TCAGTGAGCGGAAaaactaactgtagatgtagacattc	This paper
oGMB587	Barcode 3 reverse (oGMB282 site)	NGS sequencing	TCAGTCTATTCGTaaactaactgtagatgtagacattc	This paper
oGMB293	Generate runx1+23 lifeact-mCherry, forward	Gibson cloning	tgctttcgaatttcttgatcaaatctgcgacacccatgggatgtctgtttctgaggttgc	This paper

oGMB294	Generate runx1+23 lifeact-mCherry, reverse	Gibson cloning	gatcaagaaattcgaaagcatctcaaaggaagaagtgagcaagggcgaggag	This paper
	stab2 reverse†	WISH probe synthesis	AAAGAGAGCTGCACCGACT	Hagedorn *et al, Developmental Cell,* 2023
	stab2 forward*	WISH probe synthesis	TTGTGGATTACGGGGTTCGG	Hagedorn *et al, Developmental Cell,* 2023
	stab1 reverse†	WISH probe synthesis	CGCCGTTCTATAATGCACCG	Hagedorn *et al, Developmental Cell,* 2023
	stab1 forward*	WISH probe synthesis	AAGGCGTACTATGTCCTCAGGC	Hagedorn *et al, Developmental Cell,* 2023
	mrc1a reverse†	WISH probe synthesis	ACGGCATTCCACAAACCAGA	Hagedorn *et al, Developmental Cell,* 2023
	mrc1a forward*	WISH probe synthesis	GTGTCCCCTCATCAATGCCA	Hagedorn *et al, Developmental Cell,* 2023
oGMB244	*dab2* reverse†	WISH probe synthesis	gcacaggaatacttgcatagaggaa	This paper
oGMB243	*dab2* forward*	WISH probe synthesis	cctcggggagaaggagagta	This paper
oGMB406	*sele* reverse†	WISH probe synthesis	CATGGCTGCACATCACTGTC	This paper
oGMB405	*sele* forward*	WISH probe synthesis	AAGCAAGGGATCTCACCACA	This paper

*The T3 sequence CATTAACCCTCATAAAGGGAA was added to the 5' end of each forward primer for WISH probe synthesis

†The T7 sequence TAATACGACTCACTATAGGG was added to the 5' end of each reverse primer for WISH probe synthesis.

Table S2 | Plasmids

Plasmid name	Description	Source	Notes
pGMB56	lifeact-mCherry driven by runx1+23 enhancer and minimal promoter	This paper	Used to generate *Tg(runx1:lifeact-mCherry)*
pZSI3	hLNGFR-p2A-mCherry driven by runx1+23 enhancer and minimal promoter	This paper	Construct from which pGMB56 was generated using Gibson cloning
GW runx1+23-hLNGFR-mly7GFP	hLNGFR driven by runx1+23 enhancer and minimal promoter	Wattrus & Zon, Zebrafish, 2020[85]	Construct from which pZSI3 was generated using Gibson cloning

Table S3 | RNAseq FPKM values from selected hematopoietic genes

Name	Gene ID	*runx(-)cd41(+)*	*runx(+)cd41(+)*	*runx(+)cd41(-)*	Chromosome	Gene start	Gene end	Strand
runx1	ENSDARG00000087646	1.78093232	6.12817683	0.5393557	1	1362932	1402303	-
itga2b	ENSDARG00000018687	0	27.8554782	0.15943671	3	21350509	21382491	-
myb	ENSDARG00000053666	4.56910884	30.9095758	0.79415534	23	31815423	31830043	+
gata2b	ENSDARG00000009094	4.44914243	15.1279357	0.85745142	6	40794015	40803800	+
ptprc	ENSDARG00000071437	6.48797882	14.5598439	1.7320793	22	22977268	23067859	-
blf	ENSDARG00000043126	21.9869739	356.173419	55.6438925	22	9917007	9923692	+
cebpa	ENSDARG00000036074	69.6925813	106.935946	49.3083478	7	38084986	38087865	-
gata1a	ENSDARG00000013477	0.12575285	9.78311668	31.2481491	11	25411914	25418856	-
hbbe2	ENSDARG00000045143	0	6.85073268	1398.89306	12	20349865	20350629	-
hbbe1.1	ENSDARG00000113599	70.7113449	2229.80796	100811.873	3	55114097	55114874	+
hbae3	ENSDARG00000079305	46.7206209	1351.7368	97094.508	3	55146418	55147788	-
cahz	ENSDARG00000011166	4.18922798	85.8433489	6908.42985	2	29477383	29485408	-
spi1b	ENSDARG00000000767	56.4962101	75.6804271	16.3035788	7	32644673	32659353	-
mpeg1.1	ENSDARG00000055290	2.15145279	8.49601485	3.72757449	8	29710110	29713621	-
mfap4	ENSDARG00000090783	16.9606702	66.2654009	20.0949865	1	59090583	59092056	+
irf8	ENSDARG00000056407	3.20708921	11.9951888	2.71051423	18	30567945	30573849	+
mpx	ENSDARG00000019521	256.767364	204.546763	41.0180443	10	7778870	7785945	-
lyz	ENSDARG00000057789	5.68244408	17.9837621	2.18425643	24	24035863	24038800	-
cpa5	ENSDARG00000021339	249.064635	288.173385	51.9479668	25	18459924	18470695	-
mpl	ENSDARG00000039222	0.11192271	3.55822323	0.10168744	6	33949280	33980881	-
rag1	ENSDARG00000052122	0.41290322	1.32631978	0.30894167	25	9003230	9009902	+
rag2	ENSDARG00000052121	0.44224494	1.91139192	0.4018019	25	9010425	9013963	-
ikzf1	ENSDARG00000013539	14.725027	42.0403549	19.5771054	13	15957508	15994472	-
